# Frequency of Dental X-ray Diagnostics in Children and Adolescents: What Is the Radiation Exposure?

**DOI:** 10.3390/diagnostics13030394

**Published:** 2023-01-20

**Authors:** Christoph-Ludwig Hennig, Ina Manuela Schüler, Rebecca Scherbaum, Rika Buschek, Marcel Scheithauer, Collin Jacobs, Hans-Joachim Mentzel

**Affiliations:** 1 Department of Orthodontics, Center of Dental Medicine, Jena University Hospital, An der Alten Post 4, 07743 Jena, Germany; 2 Section Preventive Dentistry and Pediatric Dentistry, Department of Orthodontics, Center of Dental Medicine, Jena University Hospital, An der Alten Post 4, 07743 Jena, Germany; 3 Section of Pediatric Radiology, Department of Radiology, Jena University Hospital, Am Klinikum 1, 07747 Jena, Germany; 4 Radiation Protection, Center for Health and Safety Management, Jena University Hospital, Am Klinikum 1, 07747 Jena, Germany

**Keywords:** dental X-ray diagnostic, frequency of dental X-ray by children, orthodontic X-ray diagnostic

## Abstract

Children are exposed to ionizing radiation through radiographs during their development for various reasons. At present, there are no officially valid reference values for dental X-rays in children and adolescents for dental X-ray diagnostics. This study retrospectively examined 9680 extraoral dental radiographs in pediatric patients between 2002 and 2020. The aim was to analyze the radiation doses in pediatric patients, which indications were used, and whether there were specific age and gender differences. The evaluation showed that radiation doses were considered low, with dose area products of 2.2 cGy × cm^2^ for a lateral cephalogram, 14 cGy × cm^2^ for an orthopantomogram (OPG), and 45 cGy × cm^2^ for cone beam computer tomography (CBCT). This corresponds to an effective dose of 1.5 μSv for a lateral cephalogram, 7 μSv for an OPG, and 33.8 μSv for CBCT. Of the 9680 images, 78% were orthopantomograms, and only 0.4% were CBCT images. OPG has become more important over the years, as reflected in the indication. Approximately one-third of all extraoral exposures are orthodontic indications. Overall, the indications were similar for both genders. According to the dental indications, boys were X-rayed slightly more frequently than girls (54.5–45.5%). A future publication of dose guide values and corresponding guidelines is of high priority.

## 1. Introduction

Children and adolescents are particularly sensitive to ionizing X-rays. The consequences of radiation can be far-reaching and can affect the rest of their lives. Therefore, a sensitive approach to radiation is necessary, especially for children and adolescents. The risk of radiation damage is conditioned by various factors. An important role is played by the division rate of the cells, which is higher the younger the patient. Furthermore, the water content of children’s tissue is significantly higher, meaning that proportionally more radiation is required for the same tissue layers. In addition, sensitive radiation regions are closer together the smaller and more compact the patient’s body is. Consequently, these areas are usually located within the radiation field. The distribution of red bone marrow is also different in children compared with the adult body. In infants, for example, it is present in all areas, including the skull, which is much more important in percentage terms than in adults. Therefore, the risk is correspondingly high. In general, the probability of late effects increases with the length of life still to be expected [1,2]. Therefore, the younger the patient, the higher the risk of radiation damage. 

In dentistry, radiological imaging using X-rays is an important diagnostic procedure. The first image of teeth was made as early as 1896 by Friedrich Otto Walkhoff [3]. Since then, there have been several modernizations and extensions to the use of X-rays in the medical field. Therefore, three-dimensional imaging techniques using ionizing radiation, such as computed tomography and digital volume tomography, are already commonplace, and X-rays have become almost indispensable for adequate dental treatment [4]. Many diagnoses are only confirmed or even made with the help of these procedures. For example, the clinical suspicion of proximal caries can be confirmed, or the need for orthodontic treatment can be determined using a cephalometric analysis with a lateral cephalogram.

The majority of dental X-rays in children and adolescents are taken in the field of pediatric dentistry and orthodontics. Dental X-ray procedures are divided into two categories: intraoral and extraoral imaging techniques and two- and three-dimensional procedures. The present study refers exclusively to extraoral overview radiographs taken in two dimensions, such as orthopantomography (OPG) and lateral cephalograms, or in three dimensions, such as digital cone beam computer tomography (CBCT). OPG is used as a dental overview image in pediatric dentistry for caries diagnostics and for assessing anatomical structures in the tooth, mouth, and jaw regions. OPG and lateral cephalograms together are used for orthodontic diagnostics to establish an appropriate treatment plan and to verify the success of treatment [5]. However, if the information provided by these two-dimensional procedures is insufficient, a three-dimensional image is obtained using CBCT. In children and adolescents, its field of application mainly includes planning surgeries related to impacted teeth, cleft lip and palate, and skeletal discrepancies, in addition to orthodontics [6]. In older ages, the range of indications is mainly extended to implantology [7].

This study aimed to investigate the number and dose values of dental X-ray examinations in children and adolescents at the University Hospital in relation to the modalities of OPG, lateral cephalograms, and CBCT from January 2002 to July 2020. The aim is to determine both the dose area product of a single X-ray examination and the resulting effective dose for the patient and, simultaneously, to illustrate how this should be assessed compared to other radiation exposures. The number of X-ray examinations was analyzed both on an annual and modality basis. In addition, the indications were examined more closely to show whether they have changed over the years. Individual indications were analyzed for age peaks.

## 2. Materials and Methods

The Radiology Information System (RIS) of i-SOLUTIONS Health GmbH (RadCentre, Mannheim, Germany) and the Picture Archiving and Communication System (PACS) of GE Healthcare were used to collect the data. The data were extracted from the above programs by the University Hospital Computer Center. The patient base included all children and adolescents aged 0–18 (up to age 18) who had a radiograph taken between 1 January 2002 and 31 July 2020.

The following patient parameters were used as additional filters: identification number, hospital information system identification number (HIS ID), pseudonym with the date of birth, and gender. Examination date, doc type, room/device, dose area product, indication, and modality were also filtered out. The above information was compiled on the modalities of OPG, lateral cephalogram, and CBCT and formatted into an Excel spreadsheet. Extraoral images from OPG, lateral cephalogram, and CBCT were evaluated.

Subsequently, the values for the dose area product of the individual modalities were determined uniformly and supplemented. Using the dose values, an effective dose for the patient could be determined using the following formula:

**ED [mSv] = DAP [Gy × cm^2^] × CF skull [mSv/Gy × cm^2^]** (1)

(ED = effective dose; DAP = dose area product; CF = conversion factor).

To determine suitable conversion factors (CFs), the work of Gosch et al. (2007) [8] from the University Hospital Leipzig was used as a reference [8]. Table 1 summarizes the CFs for conventional X-ray skull examinations as a function of tube voltage in different planes (Table 1). Through this, suitable values for the extraoral X-ray procedures for lateral cephalogram, OPG, and CBCT were determined. As the lateral cephalogram has a lateral projection at a tube voltage of 73–77 kV, an averaged factor between 70 kV and 80 kV was formed from Table 1:

CF = (0.062 + 0.076)/2 = **0.069** mSv/(Gy × cm^2^). (2)

In the case of OPG and CBCT, radiation exposure occurs correspondingly from several viewing angles due to the rotation around the patient. This must be taken into account in the CF. 

For OPG, the tube voltage is 64–69 kV at the University Hospital (64 kV for an OPG child, 69 kV for all other programs). To establish a uniform CF for this modality, the value between 60 kV and 70 kV was averaged from the table. The projections resulting from the slightly more than half-ellipsoidal movement were also considered (posterior–anterior, lateral twice). Consequently, the following calculation was applied according to the recommendations of the medical physics expert:

CF = (0.034 + 0.044 + 0.049 + 0.062 + 0.049 + 0.062)/6 = **0.050** mSv/(Gy × cm^2^). (3)

Due to the tube voltage of 85 kV in CBCT, an averaged factor was used, also considering the X-ray planes due to the (half) circular motion (anterior–posterior, lateral twice). The result is as follows:

CF = (0.055 + 0.065 + 0.076 + 0.088 + 0.076 + 0.088)/6 = **0.075** mSv/(Gy × cm^2^). (4)

Statistical analysis was performed with IBM SPSS Statistics 27 using descriptive statistics as well as absolute and relative frequency distributions for categorical characteristics, such as age, gender, year, or indication. Mean models, with patient age as the dependent variable, were also used. Patient age was used as the outcome variable combined with HIS ID, which was individually assigned to each patient, as a random effect in linear mixed models (random effects model). Furthermore, cross-tabulations were conducted to better elicit associations. Multiple tests were revised using the Bonferroni correction.

## 3. Results

**Figure 1:**
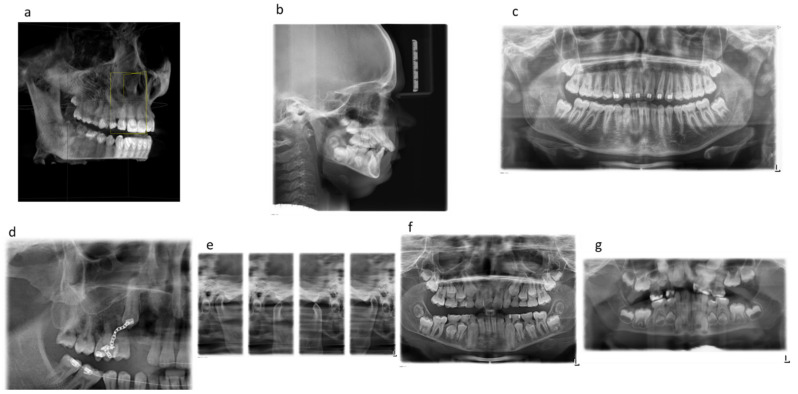
Shows examples of the extraoral dental X-ray images analyzed (Figure 1). Examples of the extraoral X-ray images analyzed: (**a**) CBCT, (**b**) lateral cephalogram, (**c**) OPG standard, (**d**) OPG without TMJ left and half OPG image, (**e**) OPG TMJ program, (**f**) OPG without TMJ right and left, (**g**) OPG child.

Figure 1 shows examples of the extraoral dental X-ray images analyzed (Figure 1).

### 3.1. Dose Values

The determination of the documented dose area product for OPG, lateral cephalogram, and CBCT X-ray examinations in children and adolescents at the University Hospital and effective doses after conversion from mSv to μSv resulted in the following values (Table 2):

### 3.2. Frequency of Dental Radiographs in Children and Adolescents per Year 

**Figure 2:**
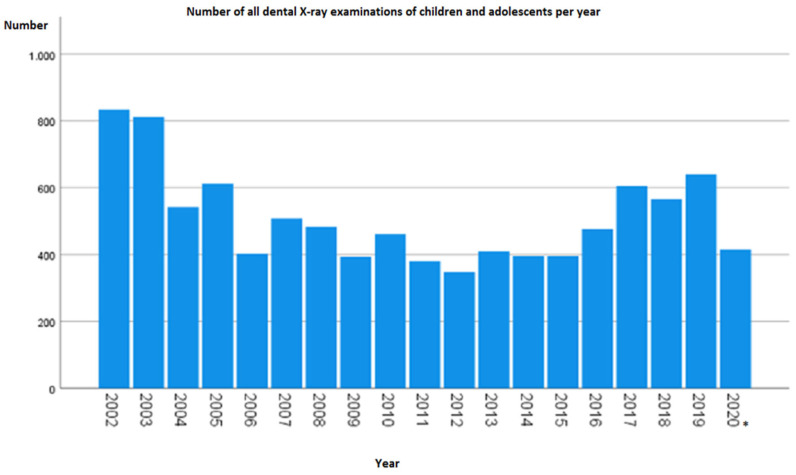
Number of dental X-ray examinations of children and adolescents at the University Hospital per year (* in the year 2020 between 1 January 2020 and 31 July).

From 1 January 2002 to 31 July 2020, a total of 9.680 dental radiographs were taken in children and adolescents in the form of OPG, lateral cephalogram, or CBCT. The analysis showed that the number of yearly radiographs varied over time (Figure 2). In 2002, more than twice as many extraoral radiographs were taken (834) as in 2006, with a minimum of 348 extraoral radiographs 10 years later (2012). Thereafter, a quantitative increase was evident. It should be noted that 2020 was recorded only until July.

As shown in the simplified modality representation, panoramic radiographs were performed most frequently among the three overview modalities in the abovementioned period. A total of 7546 (78%) orthopantomograms and 2097 (21.7%) lateral cephalograms were recorded, but CBCT was used only 37 times (0.4%). 

OPG was applied more frequently in percentage terms over the period. When the ratio of modality frequencies was compared within a year, a decrease in lateral cephalogram abundance and an increase in OPG abundance were evident. For example, at the beginning of the survey in 2002, 30.6% of the 834 images were lateral cephalograms, and 69.4% were OPGs. By contrast, in 2019, only 14.1% were lateral cephalogram images, and 84.8% were OPG images. CBCT was first used in children and adolescents at the University Hospital in 2010, when the fewest procedures were performed at two exposures (0.4%). Subsequently, the frequency varied and reached its maximum in 2019 at seven exposures (1.1%).

### 3.3. Average Patient Age per Dental X-ray Diagnosis and Indication

On average, the children who underwent CBCT scans were the oldest (12.8 years, based on a simplified modality representation). Lateral cephalogram images accounted for a lower average of 0.2 years (approximately 2.5 months). The average age for OPG was the lowest at 11.9 years. However, several different imaging options were offered. When all these findings were considered, a wide range of ages for orthopantomograms was obtained. On average, the patients were 12.7 years old for the standard OPG program but were almost three years older for the OPG temporomandibular joint (TMJ) program. Children were the youngest in the OPG-child admission type at 5.5 years and OPG-without TMJ right at 9.6 years. 

Regarding gender, boys were examined for the first time using CBCT at 5 years, approximately three years earlier than girls (8 years). Overall, girls, on average, were slightly older than boys for all imaging types (Table 3). The following were exceptions: OPG without TMJ of only one (jaw) quadrant, with an average age of under 13 years for both genders, and lateral cephalogram, with girls being slightly younger at 12.5 years than boys at 12.7 years. 

The youngest patient age for dental X-ray diagnostics was found in the indication caries diagnostic. The average age of the children was 9.2 years. They were slightly older, on average, for orthodontic imaging (12.7 years), and the oldest for X-rays related to prosthetic dentures (15.7 years), which was the aplasia case by five patients. On average, the female patients were older, except for the indications of caries diagnosis, orthodontics, and trauma (*p* = 0.008).

Approximately one-third of all extraoral X-rays due to orthodontic treatment in children and adolescents were ordered and performed from January 2011 to July 2020. About 18% of this number was conducted to inspect the dental system. The images were taken with respect to an endodontic therapy (two OPG images) or a prosthetic denture (five OPG images). Table 4 shows the frequency distribution of the indications for dental X-rays in children and adolescents.

**Figure 3:**
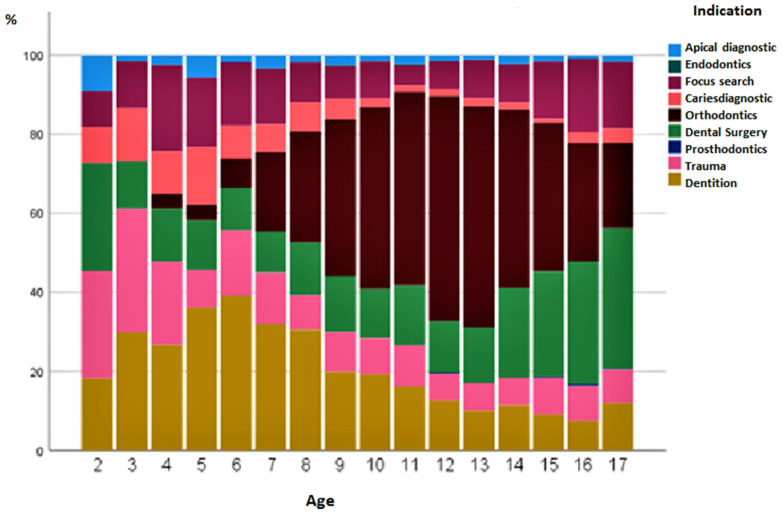
Distribution of dental indications for radiography from 1 to 18 years of age.

**Figure 4:**
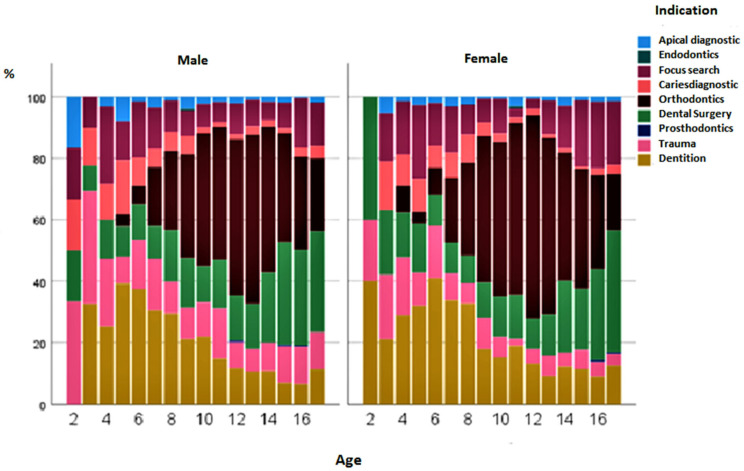
Gender distribution of dental indications for radiographs from 1 to 18 years of age.

Figure 3 shows the percentage changes in the status of the indications for over 18 years of life. No extraoral images were taken in children under 2 years of age. Orthodontics, as an indication, reached its peak at the age of 12–13 years. Subsequently, oral, maxillofacial, and facial surgery with accompanying operations increased in frequency. X-rays for the diagnosis of dental implants were carried out, especially in the first decade of life. The indication for extraoral examinations in the context of caries diagnosis at the age of 10–15 years was less frequent in terms of percentage (Figure 3). Surprisingly, girls were already examined at 4 years of age with the aid of extraoral images for orthodontic treatment. The questions were mainly related to the initial/final diagnosis, with an assessment of the tooth position and growth pattern, and to the tooth status, focusing on impacted/misplaced/missing teeth. In boys, the indication for orthodontics was first made at 5 years of age. Over the years, boys had a higher percentage of admissions for trauma (12.5%) than girls (7.2%) (*p* < 0.001). If the frequency of indications was compared across genders, it could be seen that all indications were descriptively more frequent in boys than in girls. There were also significant differences in frequency among male patients for the indications apical diagnostics (*p* = 0.047), caries diagnostics (*p* = 0.007), dental surgery (*p* < 0.001), trauma (*p* < 0.001) and prosthodontics (*p* = 0.004) (Figure 4).

In addition to the relative frequency of gender, the finding of increased trauma-related X-rays in boys was confirmed when the absolute number was considered in the cross-gender comparison. Boys were X-rayed 322 times, whereas girls were X-rayed only 148 times.

The distribution of indication frequencies by gender was predominantly comparable for boys and girls. A conspicuous feature was the orthodontic indication in girls, which accounted for 36.4%, a higher percentage of all indications than in boys (31.7%) (Figure 4). 

In the gender comparison, boys were X-rayed more frequently than girls. Statistically, 5–10% more dental X-rays were taken in boys yearly. Only 2020 showed a deviation. However, this value had limited significance, as the dataset only extended to 31 July 2020, not to the end of the year. Overall, there were 874 more exposures for boys. 

## 4. Discussion

For the final comparison, the calculated CFs were compared with the values from the literature. Looe et al. examined the CF for lateral cephalogram images (taking into account the recommendation of the ICRP) in 2007 and the CF for OPG in 2008 [9,10]. The CF for CBCT was addressed by Mah et al. (2021) [11]. All three papers reached comparable results for CFs for extraoral radiographic procedures (lateral cephalogram = 0.042–0.149 mSv/(Gy × cm^2^); OPG = 0.008–0.132 mSv/(Gy × cm^2^); CBCT = 0.035–0.31 mSv/(Gy × cm^2^)). The OPG–KF from Looe et al. was the basis for the 2018 study of Schwabl from Graz. The investigations and calculations on OPG images contained therein showed an effective dose of 10–14 μSv for the Promax 3D Max X-ray unit in adolescents and 39–54 μSv for the Orthophos XG 3D [12]. Furthermore, Schwabl compared his values with those of Ludlow et al. (2015), who examined 519 and 743 publications from PubMed and EMBASE, respectively, which was reduced to 20 studies later. In this study, the effective child dose was 13–769 μSv for the large or medium field of view (FOVs) (>15 cm height/10–15 cm height) and 7–521 μSv for small FOVs (<10 cm height) [13].

The determination of dose exposure values showed that they were very low in relation to other typical radiographic procedures [14]. For example, according to the Federal Office for Radiation Protection, the effective dose for an abdomen X-ray is significantly higher at 0.4 mSv [15]. This is approximately 60 times that of a standard OPG exposure, which was only 0.007 mSv, according to the data from our study. According to the German Federal Office for Radiation Protection (2018), dentistry accounts for the largest average share of radiographs in the course of a patient’s life (39%) but accounts for the smallest share of the collective effective dose (0.3%). Moreover, the natural radiation exposure of an individual in Germany starts at 2.1 mSv per year [15]. For comparison, one could perform 300 standard OPG exposures until this dose is reached. Consequently, the radiation exposure from dental X-rays can be considered low compared to other radiation sources and does not represent an enormous radiation exposure. However, it has to be considered that each exposure increases the stochastic risk of cancer development, even if the dose of the single exposure is considered low [16]. Still, the number of extraoral radiographs per each patient individually and the resulting increasing cumulative dose were not explicitly investigated and considered in the present work. 

This study aimed to analyze the extraoral radiographic procedures of children and adolescents up to 18 years of age for various aspects in a university dental radiology department from January 2002 to July 2020. The radiation exposure, frequency distributions, and different indications were evaluated in more detail. To the best of our knowledge, there are only a few studies that examined the topic and number of dental extraoral X-ray procedures in children and adolescents. 

A Finnish study from 2016 showed that extraoral radiographs in 7–12-year-old children accounted for the predominant share of dental radiographic examinations, with indications of orthodontics taking up the largest share at 95% [17,18]. In our study, extraoral radiographs were most frequently indicated by orthodontic issues, but orthodontics accounted for only about one-third of these. Our evaluation revealed that orthodontic radiographs were most frequently taken in terms of percentage at the age of 12–13 years. This seems plausible, as all permanent teeth (except wisdom teeth) have already erupted by this time.

Other indications in the 2016 Finnish study by Pakbaznejad Esmaeili et al. were caries diagnosis, trauma, infections, postoperative evaluations, and TMJ pain [17,18]. This correlated overwhelmingly with the present results. Therefore, preoperatively, extra-oral images were prepared in 878 cases (19%) and in 37 cases (4.2%) in connection with CBCT images. This radiographic modality is usually performed prior to more complex surgical procedures, for example, to obtain a better assessment of the access route (oral/vestibular) and adjacent anatomical structures of displaced/retained teeth or to prepare and perform interdisciplinary operations in the best possible way as part of oral surgery–orthodontic treatment [19,20,21,22]. In individual cases, or predominantly in adults, CBCT is useful for the perioperative planning of implants. Its main advantage is the possibility of measuring bone volume [7,23]. 

In our study, extraoral images were performed less frequently in caries diagnostics at 10–15 years than at earlier ages. The reason for this could be that younger children usually tolerate a panoramic radiograph better than intraoral radiographs, although this is the actual standard procedure for caries diagnosis in primary and mixed dentition. A Dutch study reported that it was not possible to take a bitewing radiograph for caries diagnosis by 6-year-old children in 18% of patients [24]. As a result, it has been shown that from the age of six, the intake of bitewing radiographs can be easily implemented in children. Nevertheless, early detection plays a crucial role, which is why extraoral radiographic procedures are used in given circumstances [21]. In the future, image sharpness could be improved, for example, by using multilayer imaging programs for panoramic slice images [25] so that caries lesions could be diagnosed more reliably. However, not all diagnoses require high image quality. As previously described, low quality is sufficient for detecting tooth attachment. OPG is suitable for confirming or identifying tooth number anomalies [5]. Evaluating the extent of dentition was a frequent indication for children in the first decade of life. The relative frequency of OPG examination increased in the last few years. Overall, a change in the frequency distributions of the various indications was observed over the period studied. Information about the average ages of children and adolescents for the different X-ray modalities, age peaks, and distribution of indications for overview radiographs could not be found by searching PubMed and Google Scholar in the literature (keywords: dental X-ray, children, radiography, panoramic, lateral cephalometric, CBCT, indication, age).

As is generally known, it is important to be aware that radiation can cause damage to health whenever X-rays are indicated. Until now, there have been no diagnostic reference values in Germany for dental overview images for children and adolescents in the form of OPG, lateral cephalogram, or DVT. Many studies have discussed the relationship between dental X-rays and cancer development in recent years. In particular, older studies have pointed out an increased risk of leukemia [26], thyroid tumors [27,28], and salivary gland tumors [29]. Meningiomas have also been addressed [30,31]. According to Claus et al. (2012), children who receive OPG at younger than 10 years of age have a 4.6-fold increased risk of developing meningioma during their lifetime [32]. By contrast, recent studies have revised the association between dental extraoral radiography and meningioma development, concluding that there is no direct causality [33,34].

It should be noted that the radiation dose to which the patient is exposed is proportional to the level of cancer risk but has no influence on the severity of possible cancer [16,35]. However, risk probability is not only influenced by dose but age and gender also play a decisive role. According to recent studies, the risks are considered to be slightly higher for females [36]. An older study on cephalometric radiography found that women were more likely to develop thyroid cancer, while men were more likely to develop brain tumors. The risk of salivary gland tumors was equally high [28]. A limitation of this study and all other studies is that radiation damage cannot be predicted because each individual reacts differently to radiation. Initially, only the frequency of dental X-ray diagnostics and the resulting radiation exposure in children and adolescents could be shown. At the beginning of the present work, it was assumed that there were no differences in the frequency distribution of X-ray examinations concerning gender. However, within the studied period, more boys tended to be X-ray examined than girls. Based on this, the hypothesis must be rejected.

In general, there is a twofold increased risk of suffering stochastic radiation damage below the age of 20 years and a threefold increased risk below the age of 10, regardless of gender. The risk decreases with increasing age and is negligible after the age of 80, as the latency period between radiation exposure from X-ray imaging and the probability of developing cancer, in all likelihood, exceeds the lifetime.

It is only possible to find comparative dose values for dental X-rays in children and adolescents to a limited extent because many studies use different measurement methods and units. Moreover, in vivo dose measurement is currently not possible [37]. The limiting factor of this study is that the values were not always completely stored in digital archiving programs and therefore had to be supplemented manually using analog data. In addition, the values refer to dental X-rays taken at the University Hospital using the X-ray equipment available here. It is quite probable that the use of other X-ray equipment results in different dose values for dental X-rays in children and adolescents. However, these should be within the present and shown range. The values determined for dental X-rays in children and adolescents thus provide an important indication of the number of dental extra-oral X-ray images and the radiation exposure. 

Further studies using the compiled dataset could be performed in the future. It would be interesting to analyze one child as an individual in the longitudinal course by determining how often the same patient was examined and with which dose. Thus, one could establish estimates for the cumulative dose in childhood and show how often and with which cumulative dose children are X-rayed on average in the first 18 years of life, depending on the question.

## 5. Conclusions

The evaluation of this study showed that X-ray procedures have become almost indispensable for dentistry, for children and adolescents as well. So far, however, no dose limits or reference values in dental X-rays of children and adolescents exist. The radiation dose to which a patient is exposed during extraoral X-ray procedures can be considered low in comparison to other X-ray exposures in the field of human medicine, or even to natural radiation exposure (cf. an effective dose of an X-ray of the abdominal cavity corresponds to approximately 57 OPG standard exposures; annual radiation exposure corresponds to approximately 300 OPG standard exposures) [15]. However, with the increasing number of X-ray examinations in one child, even a small single dose has to be considered.

OPG, as an extraoral radiographic procedure, was used more frequently during the period under study. In total, 78% of all overview images performed in the dental area were orthopantomograms. This was also reflected in the indications, with orthodontic issues accounting for the largest proportion (approximately one-third). In summary, the analysis showed that extraoral X-ray procedures are of great importance, especially for this specialty.

Even if there is no risk of deterministic radiation damage, the probability of stochastic damage may increase due to the frequency of the examinations. Therefore, in principle, developing a tumor due to frequent dental radiographs is possible. As children and adolescents have more radiation-sensitive organ regions and the probability of late effects increases with the length of their expected lifespan, this risk is particularly high. Thus, these indications should be critically examined. 

In the future, uniform diagnostic reference values for all radiographs in the dental field should be defined as part of quality assurance. In addition to having quality criteria and comprehensive staff training, these can ensure the adequate handling of ionizing radiation. 

## Figures and Tables

**Table 1:** Summary of the conversion factors for skull examinations, according to Gosch et al. (2007) [8].

Tube Voltage in kV	Posterior-Anterior Projection in mSv/(Gy × cm^2^)	Lateral Projection in mSv/(Gy × cm^2^)
60	0.034	0.049
70	0.044	0.062
80	0.055	0.076
90	0.065	0.088

**Table 2:** Dose area product per X-ray examination (DAP: dose area product, FOV: field of view/field size, OPG: orthopantomogram, TMJ: temporomandibular joint, CBCT: cone beam computed tomography) and effective dose per X-ray examination.

X-ray	DAP in cGy × cm	kV	mAs	sec	mAS	FOV in cm	Effective Dosis in μs
**CBCT**	**45**	85	5	6	28	25x12.5	33.8
**CBCT Max**	**40**	85	5	6	28	25x8	30
**CBCT Mand**	**30**	85	5	6	28	25x7	22.5
lateral cephalogram	2.2	73/77	14	9	126	18x24	1.5
OPG standard	14	69	15	13.8	207	15x13	7
OPG TMJ program	12	69	15	13.8	207	25x13	6
OPG without TMJ right	3	69	15	13.8	207	10x19	1.5
OPG without TMJ left and half OPG image	6	69	15	13.8	207	10x13/25x9	3
OPG without TMJ right and left	12	69	15	13.8	207	18x13	6
OPG child	7	64	8	13.8	207	16x7.5	3.5

**Table 3:** Type of dental X-ray diagnosis in relation to patient age.

Sex	X-ray Examination	Number	Average in Years	Standard Deviation	Min. Years	Max. Years
Male						
	CBCT	20	11.8	2.8	5.5	16.0
	Lateral cephalogram	1090	12.7	3.3	3.8	18.0
	OPG	4167	11.8	4.4	2.2	18.0
	Total	5277	12.0	4.1	2.2	18.0
Female						
	CBCT	17	14.0	2.4	8.0	17.3
	Lateral cephalogram	1007	12.5	3.2	2.8	18.0
	OPG	3379	12.1	4.2	2.2	18.0
	Total	4403	12.2	4.0	2.2	18.0
Total						
	CBCT	37	12.8	2.8	5.3	17.3
	Lateral cephalogram	2097	12.6	3.3	2.8	18.0
	OPG	7546	12.0	4.3	2.2	18.0
	Total	9680	12.1	4.1	2.2	18.0

**Table 4:** Frequency distribution of the indication for dental X-ray.

Indication	Frequency	Percentage
Apical diagnostic	92	2.0
Endodontics	2	<0.1
Focus search	578	12.5
Cariesdiagnostic	199	4.3
Orthodontics	1564	33.8
Dental Surgery	878	19.0
Prosthodontics	5	0.1
Trauma	470	10.1
Dentition	844	18.2
Total	4632	100.0

## Data Availability

Raw data can be made available upon reasonable request.
